# Differential sperm expenditure reveals a possible role for post-copulatory sexual selection in a lekking moth

**DOI:** 10.1002/ece3.458

**Published:** 2013-01-20

**Authors:** Nils Cordes, Arzu Yiğit, Leif Engqvist, Tim Schmoll

**Affiliations:** 1 Evolutionary Biology, Bielefeld UniversityMorgenbreede 45, 33615, Bielefeld, Germany; 2 Institute of Science, Department of Biology, Ordu UniversityCumhuriyet Campus, 52200, Ordu, Turkey

**Keywords:** *Achroia grisella*, eupyrene sperm, mating history, sexual selection, sperm allocation, sperm competition

## Abstract

Male reproductive success in the lesser wax moth *Achroia grisella* is strongly determined by pre-copulatory mate choice, during which females choose among males aggregated in small leks based on the attractiveness of ultrasonic songs. Nothing is known about the potential of post-copulatory mechanisms to affect male reproductive success. However, there is evidence that females at least occasionally remate with a second male and that males are unable to produce ejaculates quickly after a previous copulation. Here we investigated the effects of mating history on ejaculate size and demonstrate that the number of transferred sperm significantly decreased from first (i.e., virgin) to second (i.e., nonvirgin) copulation within individual males. For males of identical age, the number of sperm transferred was higher in virgin than in nonvirgin copulations, too, demonstrating that mating history, is responsible for the decrease in sperm numbers transferred and not the concomitant age difference. Furthermore, the number of transferred sperm was significantly repeatable within males. The demonstrated variation in ejaculate size both between subsequent copulations as well as among individuals suggests that there is allocation of a possibly limited amount of sperm. Because female fecundity is not limited by sperm availability in this system, post-copulatory mechanisms, in particular sperm competition, may play a previously underappreciated role in the lesser wax moth mating system.

## Introduction

Sexual selection, which is responsible for the evolution of many male reproductive characters (Andersson 1994), can be separated into two major components. Pre-copulatory sexual selection acts on traits affecting mating success, whereas post-copulatory sexual selection will influence the evolution of traits associated with male fertilization success under sperm competition and/or cryptic female choice (Andersson and Simmons 2006). Variation in male mating success caused either by female preferences or by male-male competition has long been documented, whereas inter-male variation in sperm competitiveness has been revealed more recently (Lewis and Austad 1990; Gage and Morrow 2003; Engqvist et al. 2007; Sherman et al. 2009). However, male reproductive success will be affected by both processes (Andersson and Simmons 2006) and the precise relationship between these episodes of sexual selection is not yet clear. In some species, they are positively correlated and seem to reinforce each other (Lewis and Austad 1994; Bangham et al. 2002; Evans et al. 2003; Hosken et al. 2008) whereas other studies have revealed a negative relationship (Warner et al. 1995; Danielsson 2001; Fu et al. 2001; Preston et al. 2001; Demary and Lewis 2007; Engqvist 2011). Very different evolutionary dynamics will result depending on the sign of this relationship. More studies addressing sperm competition in species with a well-established pre-copulatory component of sexual selection and vice versa are therefore essential in order to resolve this important evolutionary problem.

**Figure 1:**
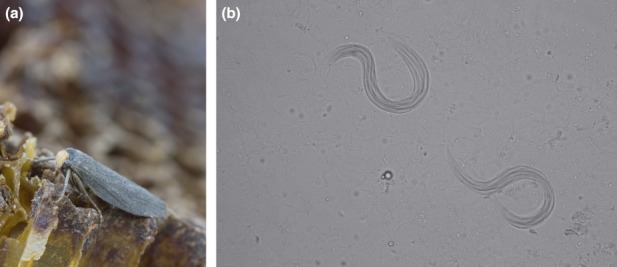
Photographs of the lesser wax moth *Achroia grisella* and its sperm: (a) Male lesser wax moth on honey comb (photograph by Nils Cordes). (b) Light microscopic picture of lesser wax moth eupyrene sperm bundles as retrieved from the female reproductive tract immediately after natural termination of copulation (400× magnification). In the background, note the filamentary apyrene spermatozoa which do not come in bundles (photograph by Arzu Yiğit).

The lesser wax moth *Achroia grisella* (Fig. 1a) is a study organism in which so far only pre-copulatory mechanisms of sexual selection have been investigated, although in great detail and very successfully. In fact, this mating system represents a model system for studying the evolutionary ecology of *pre-copulatory* female mate choice and the evolution of male secondary sexual characters (Greenfield and Coffelt 1983; Jang and Greenfield 1996, 1998; Reinhold et al. 1998; Jia et al. 2001; Brandt and Greenfield 2004; Brandt et al. 2005; Limousin and Greenfield 2009; Zhou et al. 2011). Males gather in leks to perform ultrasonic courtship songs and attract females (Spangler et al. 1984). Females then choose partners on the basis of several song components (Jang and Greenfield 1998) and allow copulation. Adult wax moths are short-lived; females live 4–7 days, males approximately 14 days in the laboratory (Künike 1930; Greenfield and Coffelt 1983). During this time, they neither drink nor feed (Greenfield and Coffelt 1983), which means that much of their activity, e.g. male courtship behaviour (Brandt and Greenfield 2004), female fecundity (Danielson-François et al. 2009), and possibly also the amount of sperm available for copulations rely heavily on accumulated reserves from larval development. Sperm is transferred in a single spermatophore during copulations typically taking around 15 min, and males resume signaling shortly after the end of copulation. Should they successfully attract another female soon after the initial copulation, they will occupy the female for several hours without transferring a spermatophore, a behaviour that is hypothesized to function as male pre-copulatory mate guarding (Greenfield and Coffelt 1983). It thus seems likely that males are unable to transfer a second spermatophore immediately after the first copulation.

Lepidoptera have two types of sperm; eupyrene, nucleate sperm used for fertilization of the eggs, and apyrene anucleate sperm whose function is not known but hypothesized to possibly play a role in sperm competition (Silberglied et al. 1984; Cook and Wedell 1999; Swallow and Wilkinson 2002). While apyrene sperm are produced exclusively at later developmental stages and may be produced throughout the adult's lifespan in some moth species, eupyrene spermatogenesis usually ends at pupation (Friedländer 1997) and adult males will typically emerge with a restricted amount of fertilizing sperm. Therefore, a relevant factor in determining the amount of eupyrene sperm per copulation is mating history, as sperm reserves may become depleted with every successful copulation and ejaculate size may decline. This has been shown across different taxa including Lepidoptera (Svärd and Wiklund 1986; Pierce et al. 1990; Pitnick and Markow 1994; Damiens and Boivin 2005; Rönn et al. 2008; Teng and Zhang 2009). This can either be interpreted as a constraint due to sperm usage in a recent mating, or it could also be considered a strategic decision: males should invest more sperm in initial copulations because each copulation can be a male's last and conserving larger amounts of sperm for future matings might be disadvantageous (Reinhold et al. 2002). In any case, a reduction in ejaculate size in successive matings has some important consequences. First, males with high mating success might risk running out of an adequate sperm supply and become ineffectual in sperm competition (Warner et al. 1995; Preston et al. 2001). Because *A. grisella* females do occasionally remate (Greenfield and Coffelt 1983), successful males would therefore invariably become less effective competitors in later copulations. Second, if female fecundity is constrained by sperm availability, preferences for fertile males should evolve, and possibly females will benefit by avoiding multiply mated males. In both these scenarios, any post-copulatory processes will have the tendency to reduce the intensity of pre-mating sexual selection. Studies on the effect of male mating history on ejaculate size in general, and the amount of eupyrene sperm in particular, might therefore contribute substantially to our understanding of sexual selection, even in those mating systems where pre-copulatory processes are supposedly or evidently prevailing. Here we therefore investigate whether *A. grisella* males transfer similar or differing amounts of eupyrene sperm in virgin *versus* subsequent nonvirgin matings, and if this potential effect is indeed a consequence of male mating history.

## Materials and Methods

### Rearing of experimental animals

We obtained experimental animals from a laboratory population of *A. grisella*, derived from approximately 100 individuals that were collected from a wild population in bee hives near Bielefeld, Germany, in October 2009. We reared the moths in 30 mL plastic cups under 12:12 h light:dark photoperiod, at 25°C, 40% relative humidity, using a breeding design which minimizes inbreeding. Larvae were supplied *ad libitum* with a food mixture modified from Jang and Greenfield (1996) and consisting of wheat, oat and maize flour (16% each), brewer's yeast (8%), glycerine (12%), honey (12%), water (12%) and used wax combs from bee hives (8%). Experimental males and females were isolated into individual cups during second to last instar to ensure virginity. Eclosion was monitored on a daily basis and we define the age of an individual at the day of its eclosion to be zero days.

### Experimental setup

In a first step, we staged experimental matings between one-day-old virgin males and randomly chosen two-day-old virgin females during a period of 4 h preceding scotophase (dark photoperiod). In a second step, we remated these males 24 h later to different randomly chosen two-day-old virgin females. We chose an interval of 24 h between matings, because male wax moths are able to successfully transfer a spermatophore approximately 8 h after a previous successful copulation (Greenfield and Coffelt 1983). To tease apart male mating history from effects of the inevitable age difference, we finally mated two-day-old virgin males to randomly selected two-day-old virgin females. We weighed individuals to the nearest 0.01 mg with a Kern 770 electronic scale (Kern & Sohn GmbH, Balingen, Germany) immediately before every copulation and measured copulation duration to the nearest 10 sec.

### Sperm bundle counts

In the lesser wax moth like in many other Lepidoptera, a spermatophore consists of solitary apyrene sperm and eupyrene sperm aggregated in bundles (256 per bundle; see Fig. 1b) (Fernandez-Winckler and da Cruz-Landim 2008); here we focused solely on eupyrene sperm. Within 5 min after copulation we dissected the female to collect the spermatophore. We chilled the female on ice for 2 min, killed the animal by decapitation with a razor blade and then cut open the ventral side, starting at the anterior end and continuing along the thorax and abdomen until the bursa copulatrix was exposed. We excised the bursa copulatrix containing the male spermatophore, transferred it to a microscopic glass slide and carefully disrupted remaining tissue with a needle tip. By adding a cover slip and applying gentle pressure we evenly spread the contents of the spermatophore over the slide. Using standard light microscopy at 400× magnification, we counted all eupyrene sperm bundles (see Fig. 1b) present on the slide twice, recording the mean value of both counts.

### Statistical analysis

All statistical tests were two-tailed and the null hypothesis was rejected at *P* < 0.05. We used R 2.14.1 (R Development Core Team 2011) for all computations. Linear mixed effects models (LME, R function *lme* in library *nlme*, Pinheiro et al. 2006) were used to test for (1) differences between virgin and nonvirgin copulations of the same males; and (2) effects of copulation duration on sperm expenditure. Male identity was included as a random effect to account for the non-independence of measurements obtained from the same individuals and to estimate between-male variation in the number of sperm bundles transferred. *P* values in the context of LME analysis refer to the increase in model deviance (compared against a χ^2^ distribution) when a term is removed from the current model and the significance of random effects was tested by removing it from the minimal adequate model after refitting this model using restricted estimation of maximum likelihood (REML). Repeatability in the number of sperm bundles transferred was calculated as the between-male variance relative to total random effects variance as estimated from the minimal adequate REML LME (Nakagawa and Schielzeth 2010). To obtain 95% confidence intervals for the repeatability estimate, we used parametric bootstrapping with 10,000 iterations as described in Faraway (2006). Due to constraints in the availability of unrelated males, the total number of 49 experimental males originated from 24 different families with on average 2.0 ± 1.5 (SD; range: one to six) brothers per family. When testing for effects of familial background on sperm expenditure, we found no effects in any of the main analyses (*P* = 0.68, 1.0 and 1.0 for the random effect of family) and we therefore did not include the respective random effects in the final models.

## Results

### Number of sperm bundles transferred

Overall, the number of eupyrene sperm bundles transferred during copulation ranged from one to 101 with a mean (±SD) of 36.6 ± 20.5 (*N* = 77 observations across male mating histories and male ages).

**Figure 2:**
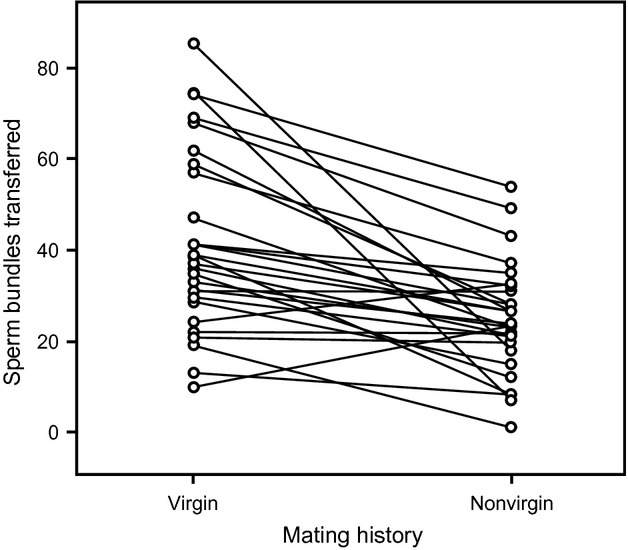
Within-individual decrease in the number of sperm bundles transferred from first (i.e., virgin) to second (i.e., nonvirgin) lifetime copulation for *N* = 28 male lesser wax moths (linear mixed effect model with male identity as random effect: χ^2^ = 18.0, df = 1, *P* < 0.001). Virgin copulations took place on day one, nonvirgin copulations on day two after eclosion.

Within individual males, the number of sperm bundles transferred decreased from 41.7 ± 3.1 (SE) to 24.5 ± 3.1 from their first (i.e., virgin) to second (i.e., nonvirgin) lifetime copulation (linear mixed effect model LME: χ^2^ = 18.0, df = 1, *P* < 0.001, Fig. 2). Conditional on the effect of mating history, we found significant between-male variation in the number of sperm bundles transferred (LME: χ^2^ = 4.1, df = 1, *P* = 0.04, see also Fig. 2). The repeatability for the number of sperm bundles transferred was 0.38 with a 95% confidence interval of 0.02–0.65.

**Figure 3:**
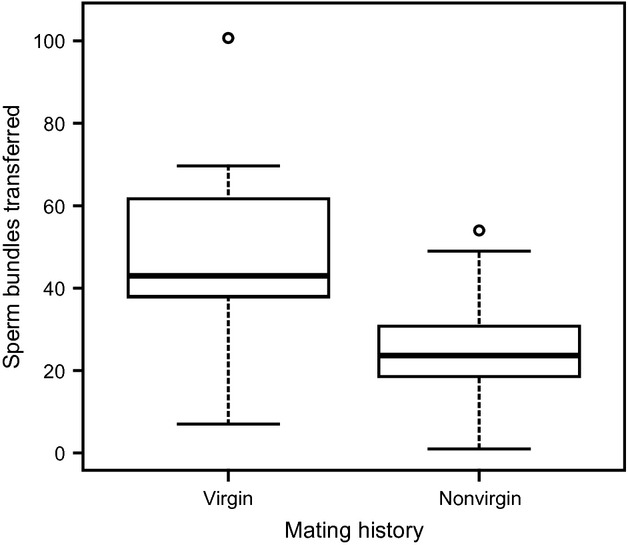
Number of sperm bundles transferred for male lesser wax moths of identical age (2 days after eclosion) during virgin (*N* = 17) and nonvirgin (*N* = 28) copulations (linear model: F_1,43_ = 18.05, *P* < 0.001). Box plots show medians, interquartile ranges (boxes) and data within 1.5 times the interquartile ranges (whiskers).

**Figure 4:**
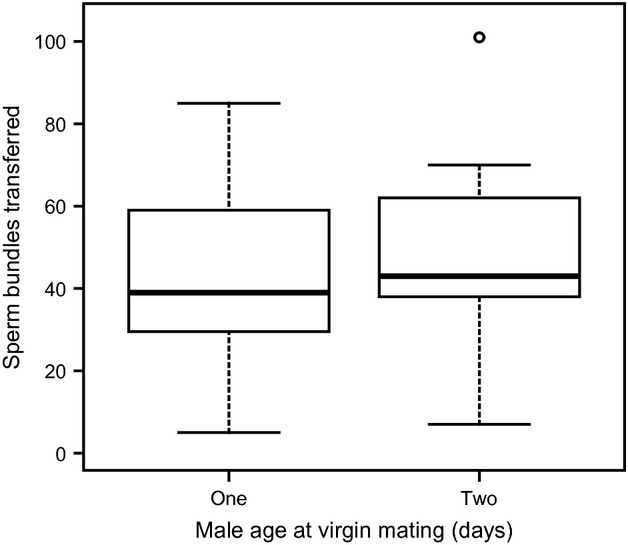
Number of sperm bundles transferred during virgin copulations for male lesser wax moths one (*N* = 32) or two days old (*N* = 17) at virgin mating (linear model: F_1,47_ = 0.68, *P* = 0.41; note that sample size for one-day-old virgin males is larger than in the paired test shown in Fig. 2 due to four males lacking a second, nonvirgin copulation). Box plots show medians, interquartile ranges (boxes) and data within 1.5 times the interquartile ranges (whiskers).

For males of identical age (i.e., 2 days after eclosion), the number of sperm bundles transferred was higher in virgin (46.9 ± 4.2) than in nonvirgin copulations (24.5 ± 3.2; linear model LM: F_1,43_ = 18.05, *P* < 0.001, Fig. 3). Furthermore, there was no difference in the number of sperm bundles transferred between virgin copulations of one-day old (41.6 ± 3.8) *versus* two-day old males (46.9 ± 5.1; LM: F_1,47_ = 0.68, *P* = 0.41, Fig. 4), indicating that indeed mating history, not the inevitable difference in age, is responsible for the decrease in sperm bundles transferred between virgin and nonvirgin copulations of the same males.

Including male or female mass prior to copulation did not improve any of the models presented above (all *P* > 0.32 or *P* > 0.24 for the effects of male or female mass, respectively, data not shown), indicating that sperm number does not depend on male size and that males do not allocate sperm in response to female quality.

### Copulation duration and sperm bundles transferred

Overall, copulation duration ranged from 120 to 2200 sec with a mean (±SD) of 898 ± 236 (*N* = 77 observations across male mating histories and male ages).

**Figure 5:**
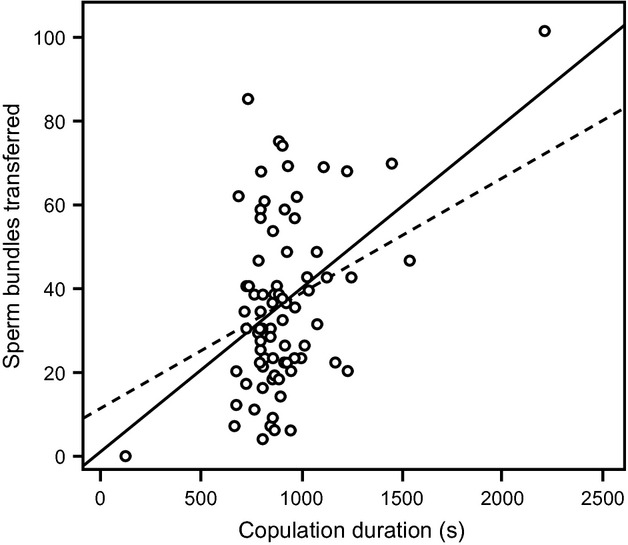
Number of sperm bundles transferred in relation to copulation duration for *N* = 77 virgin and nonvirgin copulations. The solid line represents the linear regression of the number of sperm bundles on copulation duration for all data points (linear mixed effect model with male identity as random effect: χ^2^ = 16.9, df = 1, *P* < 0.001) and the dashed line the regression when excluding the two data points with high leverage resulting from extreme copulation durations of 120 and 2200 sec (χ^2^ = 4.2, df = 1, *P* = 0.04).

The number of sperm bundles transferred increased with increasing copulation duration (LME: χ^2^ = 16.9, df = 1, *P* < 0.001, solid line in Fig. 5). This effect remained significant after excluding two data points with high leverage resulting from extreme values in copulation duration (LME: χ^2^ = 4.2, df = 1, *P* = 0.04, dashed line in Fig. 5). There was no evidence that the effects of copulation duration on the number of sperm bundles transferred differed for virgin *versus* nonvirgin copulations, indicating there were no differences in sperm transfer rate (LME: χ^2^ = 0.24 and 0.18, df = 1, *P* = 0.63 and 0.67 for the interaction between mating history and copulation duration for the full and the reduced data set, respectively).

## Discussion

We have demonstrated that the amount of eupyrene sperm transferred by male lesser wax moths during copulations decreased by over 40% from the virgin copulation to the subsequent (nonvirgin) copulation. The number of sperm bundles varied considerably and consistently among males with 38% of the variance attributable to differences between individuals.

There are several possible explanations for the observed decrease in transferred sperm over subsequent matings. Our experimental design allows us to exclude an effect of the age difference between virgin and nonvirgin copulations of the same males, because two-day-old nonvirgin males transferred significantly less sperm than two-day-old virgin males, while one-day-old virgin males did not differ from two-day-old virgin males. One could argue that males are constrained in their ability to produce another equally large spermatophore within the (experimentally controlled) period of 24 h between subsequent matings. However, when given the opportunity, males can successfully transfer a further spermatophore already 8 h after an initial copulation (Greenfield and Coffelt 1983). In the present study, males had three times longer than this to manufacture another spermatophore, so we consider it unlikely that time constraints are causing the patterns observed. Importantly, spermatophores transferred 8 h after an initial copulation are sufficiently large to guarantee fertilization of all of a female's eggs (Greenfield and Coffelt 1983). Thus, even if time constraints should exist, an explanation based on such constraints alone cannot provide a satisfactory answer as to why spermatophores from virgin copulations are substantially larger than those from nonvirgin copulations (and thereby noticeably larger than necessary for fertilization of a female's eggs).

We argue that the detected difference is probably best explained by strategic sperm allocation. Sperm production costs and/or constraints will select for male ability to prudently allocate sperm across matings (Wedell et al. 2002; Parker and Pizzari 2010). However, increasing sperm number over the level necessary for fertilization of all of a female's eggs can only be adaptive in one of two ways. Either sperm number directly affects female fecundity (e.g., Rutowski et al. 1987; Milonas et al. 2011), or sperm number is an important factor in guaranteeing male fertilization success under sperm competition. In both these scenarios, it is advantageous to enlarge ejaculates in initial matings compared to subsequent ones because males can never be certain of future mating opportunities (see Reinhold et al. 2002). This is especially relevant in a short-lived animal like the lesser wax moth. Which of these scenarios is the more likely in this case? As mentioned above, female fecundity in the lesser wax moth is *not* affected by whether she mates with a virgin or a nonvirgin male (Greenfield and Coffelt 1983), so the amount of sperm transferred by nonvirgin males does not seem to be a limiting factor for female offspring production. If, however, females mate more than once, the number of offspring a male produces depends on either quantity or quality of his sperm, setting the stage for sperm competition as a major factor to influence male fitness (Parker 1970; Simmons 2001). Reliable data on female re-mating probability in natural populations of the lesser wax moth are still lacking, but a laboratory experiment by Greenfield and Coffelt (1983) demonstrated that over 10% (4 out of 30) of females remated if given the chance and, upon dissection, contained two spermatophores – one from each male. Based on these data, which at the moment provide our best estimate, sperm competition risk (i.e., the probability that a male's ejaculate will face competition against another male's sperm) could be as high as 20–25% (∼8/34, see Parker et al. 1997). Although these results stem from laboratory-reared populations, it is likely that the opportunity to remate occurs in the field as well, where males aggregate in leks in close proximity to the bee hive (Greenfield and Coffelt 1983). Sperm competition may therefore play an important but previously underappreciated role in the mating system of the lesser wax moth.

Transferring an excessive sperm amount could thus be a way to outcompete other males' sperm and could explain the differences in sperm bundle number between subsequent matings observed here. This is the case in other Lepidoptera, in which the amount of sperm available has been shown to be allocated based on female quality, the level of sperm competition and, importantly, male mating history (Svärd and Wiklund 1986; Cook and Gage 1995; Cook and Wedell 1996; Proshold 1996; Wedell and Cook 1999; McNamara et al. 2007; Teng and Zhang 2009; Xu and Wang 2009). Hitherto, there have been no studies on post-copulatory sexual selection in the lesser wax moth; indeed, sperm competition studies in lekking insects are rare in general (but see Demary and Lewis 2007; South and Lewis 2012). In the lesser wax moth, most research has been devoted to pre-copulatory sexual selection, and between-male variation in attractiveness is well established (Jang and Greenfield 1996, 1998; Reinhold et al. 1998; Jia et al. 2001; Brandt and Greenfield 2004; Brandt et al. 2005; Limousin and Greenfield 2009; Zhou et al. 2011). It might be expected that highly attractive males must allocate their sperm reserves across more matings (Warner et al. 1995; Preston et al. 2001; Engqvist 2011), and thus sperm limitation might affect selection on traits affecting male attractiveness.

If sperm competition occurs, theory predicts that males have evolved the ability to optimize sperm production and allocation in order to be able to transfer the amounts needed to guarantee success over competitors (Parker et al. 1996, 1997; Reinhold et al. 2002). For eupyrene sperm this would lead to a picture similar to what we found in the lesser wax moth (Figs. 3). However, directional selection for optimal sperm investment should lead to a decrease in phenotypic variation as well (Parker 1993), similar to the reduction in additive genetic variance expected to occur for behavioural traits from strong precopulatory sexual selection generated by female preferences (Tomkins et al. 2004). Contrary to these expectations, *A. grisella* males vary persistently in courtship song (Jang and Greenfield 1998; Brandt and Greenfield 2004; Danielson-François et al. 2009). We demonstrate that individual male moths differed considerably in their ability to transfer high numbers of sperm during copulation as well, suggesting differences in sperm competitiveness (Lewis and Austad 1990; Simmons and Parker 1992; Dziuk 1996; Radwan 1996; Gage and Morrow 2003; Engqvist et al. 2007; Sherman et al. 2009). Thus, the between-male differences in transferred sperm bundles reported here may well translate into differences in male fertilization success. Whether males actually differ in sperm competitiveness cannot be determined from our data, and thorough paternity testing is necessary to detect and quantify the effect of sperm number on sperm competition success in the lesser wax moth. Nevertheless, given the ubiquitous importance of sperm number on sperm competition success (e.g., Parker 1982; Dickinson 1986; Parker et al. 1990; Eady 1995; Dziuk 1996; Simmons 2001; Gage and Morrow 2003; Engqvist et al. 2007), our results suggest that attractiveness and mating success alone may not guarantee fertilization success for some males, and that sperm competition may be a non-trivial source of variation in male lifetime reproductive success. The lesser wax moth could thus potentially be a valuable study system for a better understanding of the interplay between pre- and post-copulatory components of sexual selection.
